# Development and validation of a nomogram for predicting unplanned readmission within 30 days after discharge in patients with chronic heart failure

**DOI:** 10.12669/pjms.42.5.14958

**Published:** 2026-05

**Authors:** Shangran Meng, Xiandong Sun

**Affiliations:** 1 Shangran Meng, Affiliated Chifeng Clinical Medical College of Inner Mongolia Medical University, Chifeng, Inner Mongolia, China 024000. Department of Cardiology, Chifeng Municipal Hospital, Inner Mongolia Chifeng 024000; 2 Xiandong Sun, Affiliated Chifeng Clinical Medical College of Inner Mongolia Medical University, Chifeng, Inner Mongolia, China 024000. Department of Cardiology, Chifeng Municipal Hospital, Inner Mongolia Chifeng 024000

**Keywords:** Chronic heart failure, Discharge, Nomogram, Unplanned readmission

## Abstract

**Objective:**

To develop a visual assessment tool for predicting unplanned readmission within 30 days after discharge in patients with chronic heart failure (CHF).

**Methodology:**

This retrospective cohort study enrolled data from 535 patients with CHF who received treatment in Chifeng Municipal Hospital from January 2020 to September 2025. Patients’ records were divided into a training cohort (n=321) and a validation cohort (n=214) at a predefined ratio of 6:4 using simple random sampling. The Least Absolute Shrinkage and Selection Operator (LASSO) algorithm and logistic regression analysis were applied to perform feature analysis on the training cohort, which was then converted into a nomogram risk model for unplanned readmission. Comparisons were conducted using the calibration curves, receiver operating characteristic (ROC) curves, and decision curve analysis (DCA).

**Results:**

The incidence of unplanned readmission within 30 days was 19.1% (102/535). Seven identified independent risk factors for predicting unplanned readmission included age, chronic kidney disease (CKD), anemia, atrial fibrillation (AF), levels of homocysteine (Hcy), length of hospital stay (LOS), and New York Heart Association (NYHA) classification. The constructed nomogram model exhibited sufficient predictive accuracy, with area under the curve (AUC) values of 0.884 (95%CI: 0.838-0.930) in the training cohort and 0.874 (95%CI: 0.814-0.933) in the validation cohort, respectively. The Hosmer-Lemeshow (H-L) test indicated good calibration of the model.

**Conclusions:**

The predictive model for unplanned readmission within 30 days after discharge in patients with CHF developed in this study demonstrates good predictive value, serving as a reliable clinical tool for risk stratification and the early identification of high-risk populations.

## INTRODUCTION

Chronic heart failure (CHF) has a high recurrence and high hospitalization rates, and is associated with a 30 days readmission rate of 19.6% that increases to almost 24% in patients hospitalized due to heart failure.^1^,^2^ About half of readmission events are attributed to cardiovascular causes (such as worsening heart failure, arrhythmia, etc.), and the rest involve various comorbidities including renal insufficiency, chronic obstructive pulmonary disease, and infections, highlighting the complexity and clinical vulnerability of post-discharge management in heart failure patients.^3^,^4^ Frequent readmissions exacerbate physical decline, deteriorate quality of life, and significantly drive up medical costs.^2^ Therefore, reducing 30 days unplanned readmissions has become a key target for healthcare quality improvement and cost control.

To address this challenge, several studies have attempted to develop predictive models for identifying high-risk patients for readmission. A risk standardization model based on Medicare claims data, which uses 37 variables, only achieved a C-statistic of 0.60 for discriminative ability, indicating that accurate prediction is difficult to achieve relying solely on administrative data.^5^ A systematic review showed that most existing readmission predictive models have poor discriminative power (C-statistic 0.55–0.65) and generally lack the inclusion of key influencing factors such as functional status and social factors, which limits their clinical utility.^6^ While a deep learning model, reported in the study by Kim et al.,^7^ achieved an area under the curve (AUC) of 0.63 in 30 days readmission prediction, this model relied on single-center data and lacked sufficient external validation, restricting its generalizability.^7^

Another study that used nursing record text for prediction, achieving an AUC of 0.85, demonstrated that unstructured data contains important information.^8^ However, these machine learning- based methods require large-scale natural language processing resources, fail to fully integrate laboratory indicators and social determinants, and are difficult to directly deploy in routine clinical settings.^8^ Despite recent advances, a predictive tool with high accuracy, interpretability, and clinical convenience has not yet been developed. This study aimed to develop and validate an individualized tool for predicting the risk of 30 days readmission in patients with CHF, specifically designed to identify high-risk individuals for intensive transitional care during the post-discharge ‘vulnerable phase’ rather than for long-term risk surveillance.

## METHODOLOGY

This retrospective cohort study enrolled patients’ records with CHF who received treatment in Chifeng Municipal Hospital from January 2020 to September 2025. The index admission was defined as the initial hospitalization for CHF that met the inclusion criteria during the study period. The follow-up period was explicitly defined as starting on the day of discharge (Day zero) and ending on the 30th day post-discharge. The primary endpoint investigated was the first unplanned readmission within this 30 days window; subsequent readmissions for the same patient within this timeframe were not counted to ensure methodological consistency.

### Ethical approval:

The ethics committee of our hospital approved this retrospective study with the number: CK20251001, Date: October 13, 2025.

### Inclusion criteria:

- Conformed to the diagnostic process and standards specified in the 2018 Chinese Guidelines for the Diagnosis and Treatment of Heart Failure.
- First hospitalization for heart failure during the study period.
- NYHA cardiac function class II~IV at discharge.
- Patients who were successfully discharged after standardized treatment.
- Aged ≥ 18 years.
- Complete clinical data.

### Exclusion criteria:

- In-hospital death or withdrawal from treatment.
- Complicated with malignant tumors of the brain, lung, liver or other organic end-stage malignant diseases.
- Major systemic diseases of the endocrine, hematological, or immune systems, including uncontrolled thyroid or adrenal dysfunction, active hematological malignancies, severe bone marrow failure, and active connective tissue diseases requiring high-dose immunosuppressive therapy.
- Lactating or pregnant women.
- Patients who had undergone cardiac surgery.

### Clinical data collection:

The data used in this study were derived from a combined database integrating information from multiple sources, including the hospital information system, laboratory information management system, picture archiving and communication system, and electronic medical records. For missing variables in the database, supplementation was conducted by manually searching hospital medical orders and medical record systems.

The included research variables, such as sociodemographic information, smoking history, drinking history, past medical history (hypertension, diabetes, coronary heart disease, chronic kidney disease (CKD), chronic obstructive pulmonary disease (COPD), anemia, atrial fibrillation (AF), pulmonary hypertension (PH)), heart rate (HR) at admission, length of hospitalization, NYHA classification, laboratory parameters, cardiac color Doppler indicators (left ventricular end-diastolic diameter (LVEDD), left ventricular ejection fraction (LVEF)), medication during hospitalization (lipid-lowering drugs, diuretics, beta blockers, angiotensin converting enzyme inhibitors / angiotensin II Receptor blockers (ACEI/ARB), antiplatelet drugs, anticoagulants}. For the indicators repeatedly tested during hospitalization, the first inspection was used. Anemia: Hb < 120 g/L (male) or < 110 g/L (female). CKD: eGFR < 60 mL/min/1.73 m² or clinical diagnosis. AF: a documented clinical history of AF or confirmation via admission electrocardiogram (ECG) and 24 hours Holter monitoring during the index hospitalization.

Regarding the primary endpoint, due to limitations in regional health data integration, readmission data were captured exclusively within our hospital system. “Unplanned readmission” was operationally defined as any unscheduled admission via the emergency department or urgent clinic within 30 days of discharge necessitated by worsening heart failure symptoms, such as exacerbated dyspnea, edema, or electrolyte imbalances. All planned rehospitalizations, including elective percutaneous coronary intervention (PCI), scheduled cardioversion or device implantation (such as CRT/ICD), and pre-planned, short-term admissions for diagnostic evaluation, were strictly excluded from the endpoint adjudication. To minimize outcome misclassification bias, a two-reviewer adjudication process was implemented. All readmission records were independently extracted and evaluated by two researchers (SM and XS), with any discrepancies resolved through discussion and consensus with a third senior cardiovascular expert.

### Statistical analysis:

All statistical analyses and data processing were performed using SPSS 27.0 (IBM Corp, Armonk, NY, USA) and R software 4.0.2 (R Foundation for Statistical Computing, Vienna, Austria; https://www.r-project.org), with the latter specifically utilized to divide the entire study cohort into a training and a validation cohort at a 6:4 ratio via a simple random sampling algorithm. The normality of continuous variables was evaluated using the Shapiro-Wilk test. Data with non-normal distribution was presented as median and interquartile range (IQR) and analyzed using the Wilcoxon rank-sum test. Categorical data were expressed as [n (%)] and compared using the chi-square test. LASSO regression was employed to screen for independent prognostic factors. Specifically, a 10-fold cross-validation was used to determine the optimal penalty parameter, with λ_min_ selected to achieve the best predictive accuracy. Prior to LASSO modeling, all continuous variables were centralized and standardized. The predictive variables selected by LASSO regression were then incorporated into logistic regression to construct a nomogram predictive model for 30 days unplanned readmission in patients with CHF. In this step, categorical variables such as NYHA class were dummy-coded using Class-II as the reference. Additionally, the logit-linearity assumption for continuous predictors was verified using the Box-Tidwell test, confirming no significant violations. The performance of the model was evaluated in both the training and validation cohorts. Model predictive efficacy was assessed using receiver operating characteristic (ROC) curves where an area under the curve (AUC) closer to one indicates better performance and calibration curves to examine the consistency between predicted and observed risks. To evaluate the clinical utility of the model, decision curve analysis (DCA) was performed to assess its benefits. A two-tailed P value < 0.05 was considered statistically significant.

## RESULTS

Among the 535 included patients, the 30 days unplanned readmission incidences were 17.8% (57/321) in the training cohort and 21.0% (45/214) in the validation cohort. The baseline characteristics of the training and validation cohorts are described in Table-I.

**Table-I:** Comparison of basic characteristics and clinical conditions between training cohort and validation cohort.

Variables	Training cohort (n=321)	Validation cohort (n=214)	Z/χ^2^	P
Age (years), median (IQR)	67 (62-70)	68 (63-72)	-0.848	0.396
Male (yes), n(%)	185 (57.6)	117 (54.7)	0.457	0.499
BMI (kg/m²), median (IQR)	22.9 (20.7-24.7)	22.5 (20.9-25.4)	-0.444	0.657
Smoking (yes), n(%)	147 (45.8)	101 (47.2)	0.101	0.75
Drinking alcohol (yes), n(%)	122 (38.0)	75 (35.0)	0.483	0.487
Hypertension (yes), n(%)	132 (41.1)	104 (48.6)	2.911	0.088
Diabetes (yes), n(%)	100 (31.2)	53 (24.8)	2.565	0.109
Coronary heart disease (yes), n(%)	181 (56.4)	116 (54.2)	0.247	0.619
CKD (yes), n(%)	110 (34.3)	85 (39.7)	1.647	0.199
COPD (yes), n(%)	81 (25.2)	61 (28.5)	0.705	0.401
Anemia (yes), n(%)	97 (30.2)	59 (27.6)	0.436	0.509
AF (yes), n(%)	123 (38.3)	77 (36.0)	0.299	0.584
PH (yes), n(%)	90 (28.0)	56 (26.2)	0.226	0.634
HR upon admission (beats/min), median (IQR)	81 (69-85)	83.5 (68-94)	-1.371	0.17
NT-proBNP (ng/L), median (IQR)	2365 (1456-3258)	2256 (1325-3120)	-1.391	0.164
Hcy (μmol/L), median (IQR)	19.4 (13.6-25.6)	20.3 (14-25.6)	-1.072	0.284
Triacylglycerol (mmol/L), median (IQR)	1.24 (0.83-1.82)	1.3 (0.84-1.88)	-0.583	0.56
TC (mmol/L), median (IQR)	3.96 (3.25-4.76)	3.85 (2.99-4.63)	-1.499	0.134
Platelet count (×10^9^/L), median (IQR)	128 (105-167)	147 (114-184)	-1.942	0.052
WBC count (×10^9^/L), median (IQR)	5.83 (5.23-7.63)	5.97 (5.26-8.04)	-1.115	0.265
Prothrombin time (second), median (IQR)	12.7 (11.7-14.4)	13 (11.3-14.5)	-1.504	0.133
Lymphocyte count (×10^9^/L), median (IQR)	1.21 (0.91-1.48)	1.13 (0.93-1.38)	-1.712	0.087
LVEDD (mm), median (IQR)	46 (42-52)	47.5 (42-54)	-0.841	0.4
LVEF (%), median (IQR)	45 (40-48)	45 (39-48)	-0.559	0.576
Low density lipoprotein (mmol/L), median (IQR)	2.14 (1.56-2.74)	2.24 (1.6-2.8)	-0.584	0.559
High density lipoprotein (mmol/L), median (IQR)	1.13 (0.74-1.53)	1.19 (0.77-1.65)	-1.489	0.137
Creatinine (μmol/L), median (IQR)	162.6 (125-254)	154 (102-220)	-2.84	0.005
Blood potassium (mmol/L), median (IQR)	4.25 (3.84-5.06)	4.47 (3.97-5.08)	-0.791	0.429
Blood calcium (mmol/L), median (IQR)	2.24 (1.74-2.69)	2.42 (1.89-2.78)	-1.657	0.098
Blood phosphorus (mmol/L), median (IQR)	1.67 (1.31-2.08)	1.78 (1.31-2.31)	-1.436	0.151
LOS (d), median (IQR)	14 (11-16)	14 (11-16)	-1.619	0.105
Medication use during hospitalization				
Hypolipidemic drugs (yes), n(%)	204 (63.6)	121 (56.5)	2.646	0.104
Diuretics (yes), n(%)	286 (89.1)	197 (92.1)	1.282	0.258
Beta blockers (yes), n(%)	213 (66.4)	129 (60.3)	2.055	0.152
ACEI/ARB (yes), n(%)	50 (15.6)	37 (17.3)	0.277	0.599
Antiplatelet drugs (yes), n(%)	215 (67.0)	152 (71.0)	0.978	0.323
Anticoagulants (yes), n(%)	152 (47.4)	88 (41.1)	2.015	0.156
NYHA classification at discharge, n(%)			2.363	0.307
II	111 (34.6)	85 (39.7)		
III	153 (47.7)	100 (46.7)		
IV	57 (17.8)	29 (13.6)		

IQR, interquartile range; BMI, Body mass index; CKD, Chronic kidney disease; COPD, Chronic obstructive pulmonary disease; AF, Atrial fibrillation; PH, Pulmonary hypertension; HR, Heart rate; NT-proBNP, N-terminal pro-B-type natriuretic peptide; Hcy, Homocysteine; TC, Total cholesterol; WBC, White blood cell; LVEDD, left ventricular end-diastolic diameter; LVEF, left ventricular ejection fraction; LOS, length of hospital stay; ACEI/ARB, angiotensin converting enzyme inhibitors / angiotensin II Receptor blockers; NYHA, New York Heart Association.

**Fig.1:**
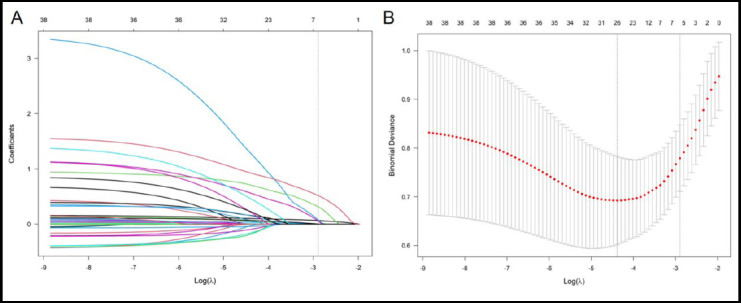
LASSO coefficient profiles for unplanned readmission in patients with CHF. A. Each curve in the figure represents the coefficient change of each variable. The ordinate denotes the coefficient value, the lower abscissa represents log(λ), and the upper abscissa indicates the number of non-zero coefficients in the model at that point. B. 10-fold cross-validation fitting followed by model selection.

First, the predictive factors for unplanned readmission were selected using LASSO regression. Variables were centralized and normalized through a 10-fold cross-validation (Fig.1). The selected predictive factors included age, complicated CKD, anemia, AF, Hcy, LOS, and NYHA classification. Second, the identified seven predictive factors were treated as independent risk variables, and a predictive model was constructed using multivariate logistic regression (Table-II). The seven predictive factors and their corresponding results were as follows: age (OR: 1.137, 95% CI: 1.078–1.198); CKD (2.614, 1.244–5.490); anemia (2.256, 1.078–4.718); AF (2.592, 1.244–5.400); Hcy (1.053, 1.016–1.090); LOS (1.100, 1.016–1.191); NYHA classification (III: 3.094, 1.126–8.497; IV: 8.956, 2.942–27.258).

**Table-II:** Multivariate logistic regression analysis of predictors selected by lasso regression in the training cohort.

Independent variables	B	OR. 95% CI	P
Age	0.128	1.137 (1.078-1.198)	<0.001
CKD	0.961	2.614 (1.244-5.490)	0.011
Anemia	0.813	2.256 (1.078-4.718)	0.031
AF	0.952	2.592 (1.244-5.400)	0.011
Hcy	0.051	1.053 (1.016-1.090)	0.004
LOS	0.095	1.100 (1.016-1.191)	0.019
NYHA classification II upon discharge		1.000 (Ref)	-
NYHA classification III upon discharge	1.129	3.094 (1.126-8.497)	0.028
NYHA classification IV upon discharge	2.192	8.956 (2.942-27.258)	<0.001

CKD, Chronic kidney disease; AF, Atrial fibrillation; Hcy, Homocysteine; NYHA, New York Heart Association.

### Nomogram model for 30 days unplanned readmission:

**Fig.2:**
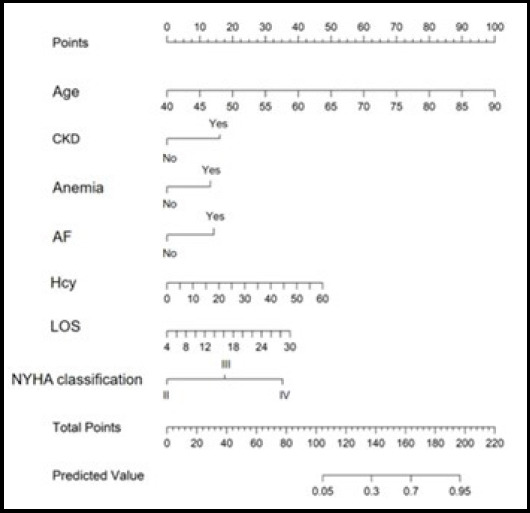
Nomogram for 30 day Unplanned Readmission. Each category of the predictive variables corresponds to a specific score. The total score is generated by summing the scores of each predictive variable, and it corresponds to the probability of unplanned readmission within 30 days.

Based on the identified seven independent risk factors, a nomogram model was constructed to predict the risk of unplanned readmission within 30 days (Fig.2). According to the nomogram, the sum of the scores corresponding to each predictive indicator was recorded as the total score. The predicted probability corresponding to the total score represents the risk of unplanned readmission within 30 days.

### Calibration and validation of the nomogram model:

**Fig.3:**
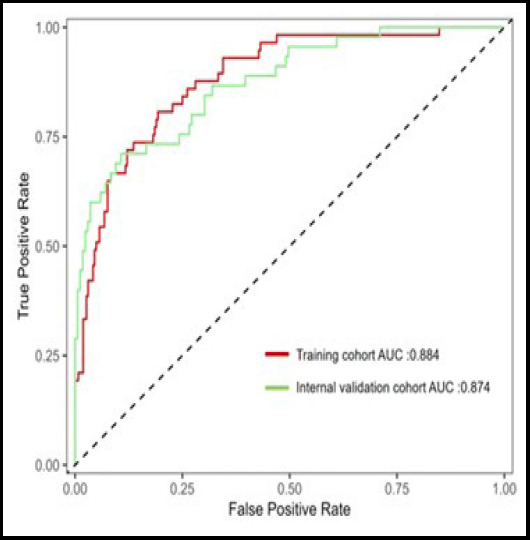
Receiver Operating Characteristic (ROC) Curves and Area Under the Curve (AUC) of the Predictive Model.

**Fig.4:**
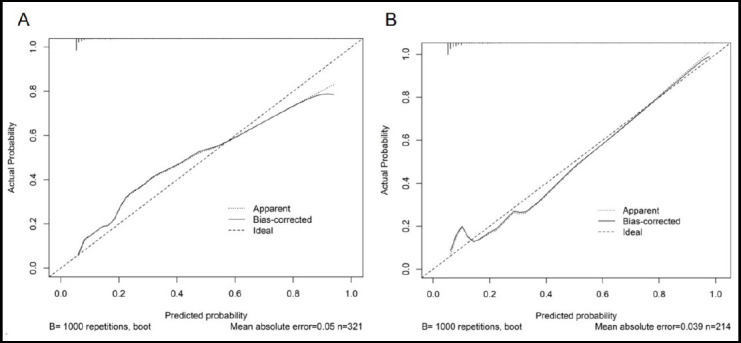
Calibration Plots of the Predictive Model. A. Calibration plot of the training cohort. B. Calibration plot of the validation cohort. The x-axis represents the predicted probability of unplanned readmission within 30 days, and the y-axis denotes the observed unplanned readmission within 30 days. The dashed diagonal line indicates the perfect prediction of an ideal model, while the solid line represents the performance of the Nomogram. A closer proximity of the solid line to the dashed diagonal line indicates better predictive performance. As shown in the figure, the predictive model exhibits good predictive ability.

**Fig.5:**
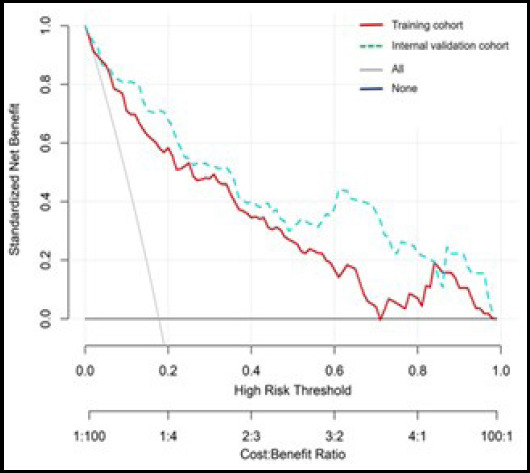
Decision Curve Analysis (DCA) of the Nomogram.

The model showed good discriminative performance in both the training and validation cohorts. In the training cohort, the apparent AUC was 0.884, and the bootstrap-corrected C-index based on 1000 resamples was 0.865. In the validation cohort, the AUC/C-index was 0.874. The Hosmer-Lemeshow test yielded non-significant results for both the training (χ²=3.823, P=0.872) and validation (χ²=10.080, P=0.260) cohorts, indicating good agreement between predicted and observed outcomes. The ROC curve in the training cohort showed good discriminative ability (AUC: 0.884; 95% CI: 0.838–0.930; sensitivity=45.6%, specificity=95.8%, positive predictive value (PPV)=70.3%, negative predictive value (NPV)=89.1%), and the C-index of bootstrapped validation (1000 bootstrap samples) was 0.865, reflecting satisfactory predictive performance. The discriminative performance of the model was validated in the validation cohort (AUC/C-index: 0.874, 95% CI: 0.814–0.933; sensitivity=40.0%, specificity=99.4%, PPV=94.7%, NPV=86.2%) (Fig.3).. In addition, calibration curve analysis demonstrated good consistency between the predicted probabilities and the observed incidence of 30 days unplanned readmission in both the training and validation cohorts (Fig.4). Decision curve analysis (DCA) confirmed the clinical utility of the model (Fig.5).

## DISCUSSION

This study aimed to construct and validate a nomogram model for predicting the risk of unplanned readmission within 30 days after discharge in patients with CHF. The results showed that the 30 days readmission rate of the cohort was 19.1%, highlighting the importance of this clinical issue and the necessity of developing accurate predictive tools. This 30 days window is widely recognized as the “vulnerable phase” in heart failure management, during which patients are at the highest risk of hemodynamic instability and clinical decompensation. Therefore, establishing risk stratification at the point of discharge is crucial for targeting early interventions. LASSO regression and multivariate logistic regression identified seven indicators including age, CKD, anemia, AF, Hcy, LOS, and NYHA classification as independent predictors. The model exhibited excellent discriminative ability (AUC > 0.87) and good calibration in both the training and validation sets, indicating that it has high predictive accuracy and clinical applicability.

To further translate this statistical applicability into actionable clinical practice, the results of the Decision Curve Analysis (DCA) (Fig.5) warrant detailed interpretation. The DCA demonstrates that the nomogram model provides a higher clinical net benefit compared to both “treat-all” and “treat-none” strategies across a broad range of threshold probabilities (approximately 10% to 80%). Given that the observed incidence of unplanned 30 days readmission in our cohort was 19.1%, a 20% threshold probability is proposed as a clinically meaningful decision point. Specifically, when the model-predicted risk is ≥ 20%, clinicians should consider triggering an intensified discharge management pathway. Actionable clinical steps include scheduling the first follow-up or tele-consultation within 7–14 days post-discharge, initiating remote symptom monitoring, providing pharmacist-led medication titration guidance, or assigning the patient to a multidisciplinary team (MDT) for prioritized care. Such risk-stratified management ensures that medical resources are precisely allocated to high-risk patients who stand to benefit most, thereby potentially reducing overall readmission rates.

Among the seven independent predictors identified in this study, age, NYHA classification, and LOS are classic clinical indicators repeatedly validated by multiple studies.^4^,^9^,^10^ Advanced age is associated with decreased physiological reserve and increased burden of multiple comorbidities, and it has been confirmed as one of the key variables for predicting heart failure readmission in numerous studies.^11^,^12^ As a marker of cardiac function status, higher NYHA classification indicates poor myocardial contractile reserve and more significant systemic hypoperfusion. Studies have pointed out that NYHA class III–IV is a strong predictor of short-term readmission in heart failure patients.^13^,^14^ LOS often reflects the complexity of the condition and the difficulty of treatment during hospitalization; studies have also confirmed that prolonged hospital stay is positively correlated with the risk of early post-discharge readmission.^4^,^15^ It is important to note that in our predictive model, variables such as LOS and NYHA class should be interpreted primarily as risk markers reflecting the overall clinical burden and biological stability at discharge, rather than direct modifiable causal drivers. Although NYHA grading is inherently prone to inter-rater subjectivity, this bias was minimized in our cohort through independent consensus assessments by two senior cardiologists based on the 2018 Chinese Guidelines. Similarly, while LOS is a composite outcome influenced by hospital-specific administrative processes, the relatively standardized clinical pathways within our single-center setting helped mitigate process-related heterogeneity. Together, these indicators provide a holistic view of the patient’s “vulnerable state” during the transition from hospital to home.

Consistent with previous studies, CKD (defined by eGFR < 60 mL/min/1.73 m^2^ or clinical diagnosis) emerged as an independent predictor of 30 days readmission in our cohort. Mechanistically, it is hypothesized that CKD is associated with sodium and water retention, as well as persistent activation of the renin-angiotensin-aldosterone and sympathetic nervous systems. These factors may potentially promote myocardial fibrosis and inflammation, thereby serving as a marker for the accelerated progression of heart failure.^4^,^16^ Another systematic review also listed renal insufficiency as a high-frequency predictor.^17^

Anemia also significantly predicted readmission, aligning with previous meta-analyses.^18^ It further impairs cardiac function during the vulnerable period via the cardiorenal-anemia syndrome: decreased hemoglobin reduces oxygen-carrying capacity, triggering compensatory hyperdynamic circulation and increased cardiac load, which in turn exacerbates heart failure.^4^

AF was also identified as an independent risk factor of 30 days readmission in this study. AF and heart failure share a complex, reciprocal pathophysiological association: the history of AF is associated with the loss of atrial auxiliary pump function, irregular ventricular rate, shortened filling time, and an increased risk of thromboembolism. These factors are hypothesized to potentially impact cardiac stability and precipitate heart failure decompensation, although further research is required to establish exact causal pathways.^19^,^20^ Clinical studies have pointed out that a history of AF is an independent predictor of readmission in heart failure patients, and a subgroup analysis showed that AF is associated with poorer prognosis.^21^,^22^ However, it should be noted that AF is a highly heterogeneous condition, and its pathophysiological impact on heart failure decompensation may vary significantly among paroxysmal, persistent, and permanent types. Furthermore, the interplay between AF and different heart failure phenotypes (HFrEF, HFmrEF, and HFpEF) warrants further prospective investigation. Due to the retrospective nature of this study, detailed subtyping of AF was not feasible, and our model currently treats AF as a uniform binary construct, which represents a limitation of our research.

A distinguishing feature of the model developed in this study from previous ones is the inclusion of Hcy, a biomarker that has garnered increasing attention in recent years. Hcy participates in the progression of CHF through multiple pathways, including inducing endothelial dysfunction, promoting oxidative stress and inflammatory responses, and stimulating myocardial fibrosis.^23^,^24^ Studies have reported that Hcy levels are negatively correlated with left ventricular ejection fraction and positively correlated with left ventricular end-diastolic diameter.^25^,^26^ In patients with acute heart failure, the risk of readmission or death within six months was significantly higher in the Hcy-elevated group than in the Hcy-normal group.^25^ These results are consistent with the findings of this study, indicating that Hcy has potential value in assessing the risk of heart failure readmission. However, some previous study models did not include Hcy as a routine predictive indicator, possibly because therapies that reduce homocysteine levels do not affect the inflammatory status of patients with cardiovascular diseases and have little impact on their cardiovascular risk.^27^–^29^ Nevertheless, this study still demonstrated the independent predictive role of Hcy in the multivariate model, suggesting that Hcy can provide additional prognostic information after adjusting for confounding factors such as renal function.

### Strengths of the study:

First, it demonstrates high methodological rigor by employing LASSO regression for variable selection, which effectively prevents overfitting, and ensures reliability through dual validation in both training and validation cohorts. Notably, during this variable selection process, classic echocardiographic parameters such as LVEF and LVEDD were included in the initial candidate pool but were not retained. This exclusion is a direct result of the LASSO regression’s selection mechanism, which handles highly correlated variables by shrinking the coefficients of redundant predictors to zero. In our cohort, their coefficients were reduced to zero earlier, suggesting limited incremental predictive value for short-term 30 days readmission. Clinically, this is largely explained by their strong multicollinearity with the NYHA classification, which integrates cardiac reserve and systemic perfusion, thereby offering greater statistical robustness in capturing the patient’s “overall vulnerability.” Second, the model exhibits excellent predictive performance with AUC values exceeding 0.87. Third, the incorporation of Hcy as a promising biomarker provides a novel perspective for assessing readmission risk in CHF. Finally, the model is presented as a visual nomogram, and all seven identified predictors are low-cost, routine clinical items, endowing it with strong clinical practicality and the potential for widespread implementation in primary care settings.

### Limitations:

First, its retrospective, single-center design and strict exclusion of complex systemic comorbidities may introduce selection bias and limit external generalizability. Furthermore, treating unrecorded out-of-hospital deaths as “no readmission” overlooks the competing risk of early mortality, potentially underestimating the true adverse burden. Second, the lack of data on post-discharge guideline-directed medical therapy (GDMT) and patient adherence introduces residual confounding. Consequently, predictors like NYHA class and length of stay primarily serve as baseline severity proxies rather than direct, modifiable targets. Third, utilizing admission laboratory values rather than pre-discharge metrics provides a standardized baseline but omits dynamic treatment response trends. Fourth, the moderate sample size (n=535) warrants larger cohorts for stabler parameter estimates. Finally, the study period coincided with the COVID-19 pandemic, which may have uniquely confounded healthcare-seeking behaviors and readmission rates. Future multi-center prospective studies integrating competing risk models and dynamic variables are needed to validate and refine this tool.

## CONCLUSION

This study successfully developed and validated a nomogram using seven easily accessible clinical indicators to predict 30 days unplanned readmission in CHF patients, demonstrating excellent discrimination, calibration, and clinical utility. By facilitating the early identification of high-risk individuals, this tool supports targeted intensive management and optimal resource allocation. Future multi-center prospective and interventional studies are warranted to confirm its generalizability and clinical benefit.

### Recommendations:

Regarding future research directions, several priorities have been identified. First, multi-center, prospective studies are required to further validate the external generalizability of the model across different regions and populations. Second, follow-up research should aim to integrate more diverse variables, such as medication adherence, psychosocial support, and novel biomarkers, to enhance the model’s completeness and precision. Third, future efforts must focus on designing clinical interventional studies based on this risk-stratification tool to evaluate whether targeted intensive management can effectively reduce readmission rates in high-risk patients, thereby achieving the transition from precision prediction to precision intervention.

### Authors’ contributions:

**SM:** Literature search, study design and manuscript writing.

**SM** and **XS:** Data collection, data analysis and interpretation. Critical review

**SM:** Manuscript revision and validation and is responsible for the integrity of the study.

All authors have read and approved the final manuscript.
